# Nutritional Composition and Odor-Contributing Volatile Compounds of the Edible Mushroom *Cantharellus alborufescens*

**DOI:** 10.3390/molecules28227516

**Published:** 2023-11-10

**Authors:** Mohaddeseh Moghaddam, Masoomeh Ghobad-Nejhad, Thomas Stegemann, Serhat Sezai Çiçek, Christian Zidorn, Majid Javanmard

**Affiliations:** 1 Department of Pharmaceutical Biology, Kiel University, Gutenbergstraße 76, 24118 Kiel, Germany; mmoghaddam@pharmazie.uni-kiel.de (M.M.); tstegemann@bot.uni-kiel.de (T.S.); serhatsezai.cicek@haw-hamburg.de (S.S.Ç.); 2 Department of Biotechnology, Iranian Research Organization for Science and Technology (IROST), Tehran 3353136846, Iran; 3 Botanical Institute and Botanic Gardens, Kiel University, Am Botanischen Garten 1-9, 24118 Kiel, Germany; 4 Department of Biotechnology, Hamburg University of Applied Sciences, Ulmenliet 20, 21033 Hamburg, Germany; 5 Department of Chemical Technology, Iranian Research Organization for Science and Technology (IROST), Tehran 3353136846, Iran; javanmard@irost.ir

**Keywords:** basidiomycete mushrooms, chanterelles, nutrients, PUFA, vitamin E, volatile substances

## Abstract

Chanterelles are one of the most highly valued wild edible mushroom genera worldwide. This work aimed to investigate the nutritional characteristics and volatile compounds’ profile of *Cantharellus alborufescens* for the first time. Proximate analysis was performed according to the Association of Official Agricultural Chemists, while the mineral contents and the volatile compounds were determined using ICP-MS and GC-MS, respectively. *C. alborufescens* had an average of 25.8% protein, 5.5% fat, 12.7% ash, and 55.9% carbohydrates, including 11.4% fiber per dw of mushroom. Further analyses of the fat and protein contents revealed high amounts of polyunsaturated fatty acids as well as monosodium glutamate-like amino acids. Linoleic acid (42.0% of fat) and oleic acid (28.6% of fat) were the major fatty acids, while leucine (1.2%) and lysine (0.9%) were the most abundant essential amino acids. The results showed that *C. alborufescens* contained 3.1 µg/g vitamin D2 and 4.9 mg/g vitamin E per dw, as well as notable quantities of macro- and microelements, such as potassium, calcium, magnesium, and iron. GC-MS analysis revealed various volatile compounds such as acetaldehyde, *n*-hexanal, 3-methylbutanal, 1-octen-3-ol, etc. In conclusion, this study supports the use of *C. alborufescens* as a food rich in fiber and vitamin E, with a suitable amount of protein and other nutrients.

## 1. Introduction

Edible mushrooms are becoming increasingly popular as a source of food for humans, especially for vegetarians and vegans. Mushrooms are the natural and non-animal source of vitamin D and contain higher amounts of proteins than most vegetables [1]. In addition to high protein and low fat contents, mushrooms also have excellent aroma and flavor properties that make them ideal components of low-calorie diets [2]. It is estimated that there are approximately 20,000 species of mushrooms on Earth; among them, more than 3000 are edible, and 200 of those are collected from the wild [3]. Chanterelles are amongst the most highly valued mushrooms, and they are harvested in the wild. In the past, chanterelles from all over the world were summarized under the scientific name *Cantharellus cibarius* Fr., which referred to nearly all prized edible yellow mushrooms with a base and a cap with radial wrinkles on the underside. In the previous decades, with the progress of molecular biology, the differences between various populations of this taxon in different geographical areas were revealed and several species were newly described [4]. So far, the nutritional and medicinal properties of only a few of these chanterelle species, in particular, of *Cantharellus cibarius*, have been studied [5,6,7,8,9,10,11]. For example, chanterelles are reported to have high levels of bioavailable vitamin D2, which is critical for bone health [12]. The pleasant smell and taste of chanterelles are partly attributed to volatile compounds, including saturated and unsaturated aldehydes and ketones [13].

Chanterelles are widely distributed in various parts of the world, including Europe, Asia, Africa, and Central America [14]. *Cantharellus alborufescens* (Malençon) Papetti & S. Alberti (Cantharellaceae) grows in calcareous soils and its distribution is mostly associated with the occurrence of Mediterranean evergreen oaks in Europe. Parad et al. (2018) also reported this species from the forests of northern Iran [15]. There are no reports on the chemical composition and nutritional value of *C. alborufescens*. Therefore, the study at hand aimed to evaluate the dietary characteristics as well as the volatile constituents of this species. Moreover, the analysis of the mineral content of *C. alborufescens* was performed.

## 2. Results and Discussion

### 2.1. Proximate Analysis

The proximate analysis was conducted in triplicate and the results are shown in Table 1. According to the results, *C. alborufescens* has an average of 90.5% moisture, 25.8% protein, 5.5% fat, 12.7% ash, and 55.9% carbohydrates, including 11.4% fiber, and around 380 kcal of energy per 100 g of dry sample.

Based on the results, the carbohydrates and crude protein content comprise the largest part of the nutrient content of *C. alborufescens*, while the fat content is low, making it suitable for healthy and calorie-restricted diets [16]. Meanwhile, *Cantharellus alborufescens* contains essential fatty acids (Section 2.2, Table 2) that can be physiologically active in a variety of ways. The protein content of this mushroom is on average more than a quarter of its dry weight (25.8%).

**Figure 1:**
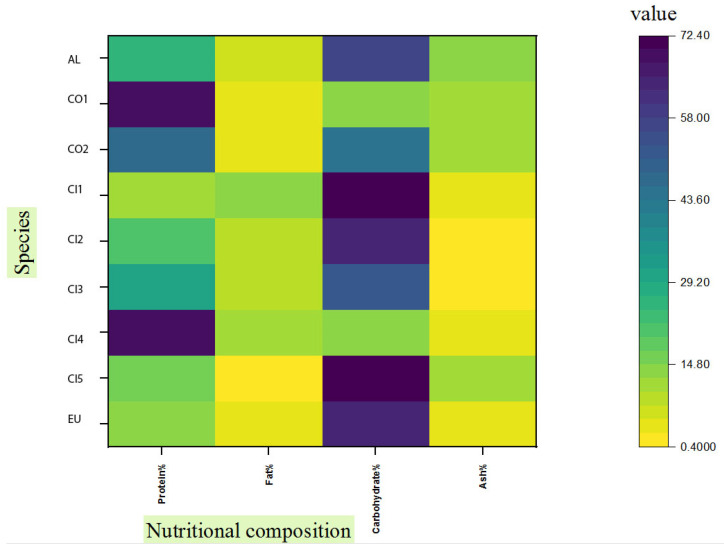
Comparison of nutrient contents (protein, fat, carbohydrate and ash percentages) in different chanterelles: (AL) *C. alborufescens*; (CO 1–2) *C. cornucopioides*; (CI 1–5) *C. cibarius*; (EU) ‘Ero ububa, purple Cantharellus’. Sources of data except for *C. alborufescens* (this study) can be found in the following references: [5,6,8,9,10,20].

In Figure 1, we summarize nutritional information on chanterelle mushrooms. Most of the data available in the literature belong to *Cantharellus cibarius*. (The values may vary for the same species due to various factors such as the stage of development and environmental conditions, especially soil and climate characteristics [17]). Despite the fact that *C. alborufescens* has a high carbohydrate content compared to closely related species, its protein content is only average. Among mushroom species, total carbohydrate content ranges from 35% to 70% dw, including digestible and indigestible carbohydrates. Non-digestible carbohydrates include beta-glucans, mannans, and non-starch polysaccharides (NSPs). Humans can benefit from these non-digestible carbohydrates as dietary fibers [18]. Fiber-rich foods contain at least 6 g of fiber per 100 g [19], and *C. alborufescens* has an average content of 11.4 g fiber per 100 g dry weight.

According to Figure 1, the ash content in *C. alborufescens* (12.7%) is higher than in other chanterelles and it is related to the presence of nutritionally important minerals (Section 2.5). The physiological and chemical characteristics of soil, along with the species of mushrooms, determine the concentrations of trace elements in the fruiting bodies [21]. Since the majority of mushroom carbohydrates are non-digestible, these polysaccharides cannot be a source of energy for humans. Nevertheless, *C. alborufescens* represents a food source with a good amount of protein, fiber, and minerals as well as a low fat content.

### 2.2. Fatty Acid Composition

The results obtained from the analyses of fatty acid contents of *C. alborufescens* are shown in Table 2. In general, the major fatty acid found in this mushroom was linoleic acid (42.0% of fat), followed by oleic acid (28.6% of fat), and palmitic acid (18.2% of fat). Besides these, eleven additional fatty acids were detected (Table 2). The amount of unsaturated fatty acids in *C. alborufescens* is higher than for the saturated ones, which is in agreement with results from related species [6,9]. Due to the high amount of linoleic acid in this mushroom, the polyunsaturated fatty acid (PUFA) content is 1.5 times higher than the saturated fatty acid (SFA) content. Since PUFAs in the diet can reduce low-density lipoprotein cholesterol (LDL-C), a high PUFA/SFA index indicates a positive effect on cardiovascular health (CVH) [22]. Linoleic acid is believed to be a precursor of compounds that contribute to mushroom flavor; *C. alborufescens* can be considered as a good food source for this essential fatty acid [2].

### 2.3. Amino Acid Composition

In this study, 16 amino acids were detected in *C. alborufescens* and the results are shown in Table 3. As part of an overall increase in protein and nitrogen intake, consumers need minor quantities of basic amino acids from biologically available sources. Mushrooms are good sources for both essential and non-essential amino acids [23,24]. The most abundant essential amino acids in *C. alborufescens* were leucine and lysine with 1.2% and 0.9%, respectively. Beluhan et al. (2011) found that lysine and threonine are the most prevalent amino acids in *C. cibarius* (5.74 and 8.98 mg/g dw) [5]. Generally, the amounts of non-essential amino acids in *C. alborufescens* were higher than those for essential ones, resulting in a 0.39 ratio. Glutamic acid was present in the highest amount (3.80%), which may especially contribute to the savory flavor of this mushroom.

**Figure 2:**
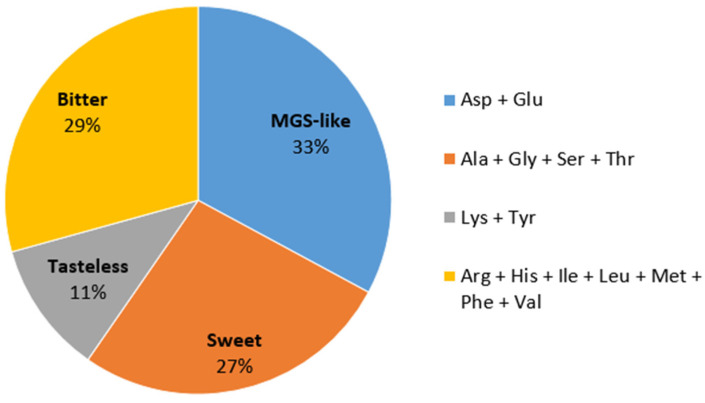
Amino acid contents in *C. alborufescens* based on taste characteristics: monosodium glutamate-like (MGS-like), bitter, sweet and tasteless. The percentage of each flavor is shown in the pie chart. Ala: alanine, Arg: arginine, Asp: aspartic acid, Glu: glutamic acid, Gly: glycine, His: histidine, Ile: isoleucine, Leu: leucine, Lys: lysine, Met: methionine, Phe: phenylalanine, Ser: serine, Thr: threonine, Tyr: tyrosine, Val: valine.

Amino acids are reported to play a role in mushroom taste, which is crucial to consumers’ acceptance. As shown in Figure 2, contents of free amino acids based on taste characteristics can be divided into four groups [25,26]. Monosodium glutamate-like (MGS-like) and sweet components are involved in the pleasant taste of mushrooms. In *C. alborufescens*, MGS-like amino acids contributed the highest amounts, followed by bitter amino acids. Apparently, the sweet tasting compounds mask the taste of the bitter amino acids well. According to the FAO/WHO (1973), every adult human needs 14 mg and 12 mg/kg body weight of the essential amino acids leucine and lysine per day, respectively. Considering the average weight of an adult to be 70 kg, each person needs 980 mg of leucine and 840 mg of lysine on average per day. By consuming 30 g of dried *C. alborufescens* per day, 360 mg of leucine and 273 mg of lysine can be obtained, corresponding to more than 30% of the daily requirement.

### 2.4. Vitamin Content

Our results showed that *C. alborufescens* contains 3.1 µg/g dw of vitamin D2 (ergocalciferol) and 4.9 mg/g dw of vitamin E (alpha-tocopherol). The amount of vitamin D2 in *C. alborufescens* in our study is higher than the values reported for *C. cibarius* (0.5, 0.84, and 1.5 µg/g dw) in the literature [12,27,28]. Phillips et al. (2011) studied the vitamin D2 and sterol contents of ten species of mushrooms, including white button, shiitake, and oyster. Among them, *C. cibarius* contained the highest amount of vitamin D2 (0.5 µg/g dw), which is probably explained by the morphology and the growth condition for the chanterelle mushrooms (more exposure to sunlight on the gills), combined with a relatively high natural vitamin D2 content [12,27].

Few papers have been published on the tocopherol content of chanterelles. Barros et al. (2008) reported 0.12 µg/g dw of alpha-tocopherol in *C. cibarius* [6]. Natural antioxidants like vitamin E play an important role in human health. The biologically active form of vitamin E is alpha-tocopherol [29]. According to the daily uptake amounts reported by the FDA in March 2020, we should consume 20 µg of vitamin D and 15 mg of alpha-tocopherol per day. Accordingly, a daily consumption of 30 g of fresh *C. alborufescens* can provide approximately the required content of vitamin E and half of the vitamin D needed by the human body.

### 2.5. Mineral Content

The concentrations of minerals in *C. alborufescens* are shown in the Table 4 and Table 5. The results show that the order of mineral contents is K > P > Ca > Mg > Na > Fe > Zn > Cu > Mn > Cr and the order of heavy metal contents is Pb > As > Cd > Hg. Potassium constituted the most abundant element and Na was the fifth. The Na/K ratio was thus less than 0.066. According to WHO recommendations, sodium intake should not exceed 2000 mg daily and potassium intake should not exceed 3510 mg daily, resulting in a Na/K ratio of ≤1.0, which is optimal for cardiovascular health [30].

The mineral contents of *C. alborufescens* compared to the daily values (DVs) reported by the FDA in March 2020 are shown in Table 4 (considering that each person consumes 30 g of dried mushrooms daily). According to the results, *C. alborufescens* can be a good source of minerals, especially potassium and iron. The updated DVs from the FDA for Cu and Cr have been reduced compared to the original values. Now, if we compare the amounts of these minerals in the mushroom with the original amounts (2.00 and 0.12 mg, respectively), consuming 300 g of fresh *C. alborufescens* per day will be harmless to humans, but according to the updated values, both the copper and the chromium contents will exceed the recommended limits.

In order to determine the heavy metals in *C. alborufescens*, the Health Risk Index (HRI) was calculated (Table 5). HRI > 1 for any metal in food indicates that the consumer population faces a health risk. In this case, the concentrations of Pb and Cd are at safe levels, but for As and Hg (and, in particular, for As), HRIs exceed the levels considered safe, which means they require monitoring. Mushroom fruiting bodies have the capacity to absorb and bioaccumulate metals and metalloids from the substrate via their mycelia. Therefore, it can be assumed that a high level of arsenic might result either from bioaccumulation (where it is preferentially absorbed from the soil with low levels of arsenic) or from “normal uptake” from soil with a high level of arsenic [31].

In the areas contaminated with heavy metals, ion bioaccumulation can occur in the mushroom fruiting bodies. Therefore, it is important to investigate the element’s contents in edible mushrooms. The study by Grzybek and Janczy in 1990 showed that the amounts of lead and cadmium in *C. cibarius* were lower than in other edible mushrooms collected at the same site [32]. Generally, heavy metal bioaccumulation by mushrooms is complex and is influenced by both environmental as well as intrinsic factors. Different species behave differently according to their physiological requirements [33]. Since we do not know the amounts of As and Hg in the mushroom substrate in the present study, a more comprehensive investigation of the heavy metal bioaccumulation in *C. alborufescens* seems warranted.

### 2.6. Volatile Compound Profile

**Figure 3:**
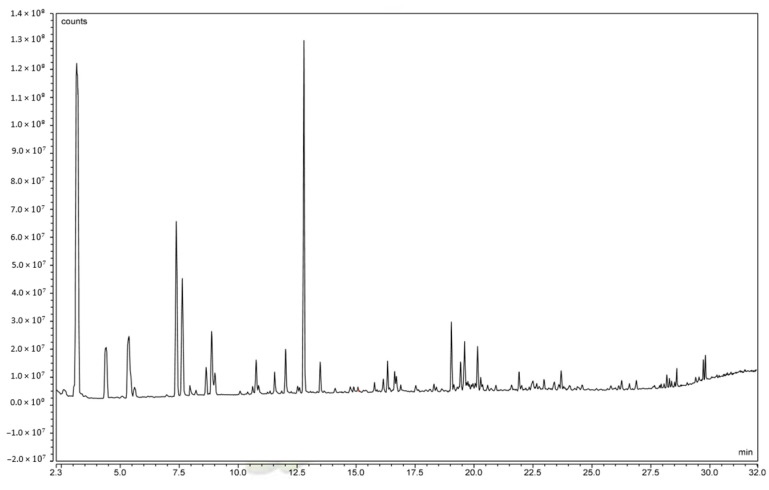
Headspace GC-MS chromatogram (time vs. abundance) of *C. alborufescens*.

An analysis of volatile and subsequent odor-determining constituents of *C. alborufescens* is shown in Table 6 and the GC-MS chromatogram is shown in Figure 3. The 30 most abundant constituents with similarity indices (SIs) of more than 750 are listed in Table 6. Contents are given relative to all constituents (total content—ToC) and to all constituents excluding water (water-free content—WfC).

Of the 30 most abundant volatile constituents found in *C. alborufescens* (Table 6), 13 compounds belong to the class of aldehydes and 6 to the class of ketones. In addition, alcohols, carboxyl esters, and nitrogen constituents were identified. Also, in terms of quantity, aldehydes represent the major compound class, with acetaldehyde, *n*-hexanal, 3-methylbutanal, 2-methylbutanal, and *n*-butanal showing water-free contents of more than 5%, only surpassed by acetone with an amount of around 28%. Aisala et al. (2019) reported volatile compounds in the *Cantharellus cibarius* and *Craterellus tubaeformis* species using the headspace solid-phase micro-extraction method. Their results generally agree with this research and show the effective role of aldehydes and ketones in the odor of mushrooms. Although, acetaldehyde was not reported in their species, and the amount of acetone was reported to be very low compared to *C. alborufescens*, while the highest amount for a compound in *C. cibarius* was *n*-hexanal [13].

Two of the main compounds responsible for ‘mushroom-like flavor’ in many species are 1-octen-3-ol and 1-octen-3-one [34]. The amount of 1-octen-3-ol alcohol (1.58 WfC%) in *C. alborufescens* is not significant; it may be be influenced by various factors such as the maturity stage, drying temperature, analysis method, and the duration of mushroom storage, in addition to the type of species [8,35]. Among N-containing compounds in *C. alborufescens*, there are three pyrazine derivatives. Rychen et al. (2016) conducted comprehensive research on the use of 22 compounds of pyrazine derivatives (including methylpyrazine, ethylpyrazine, and dimethylpyrazine) as a food flavoring for different animals; they reported that if these compounds are used in feeds at the highest level proposed, there is no safety concern for the consumer [36]. Among the volatile compounds, an aziridine derivative (methylpyrazine) was also detected here. It has been proven that the toxicity of aziridine derivatives depends on their structure and activity [37]. Aziridines have been reported to cause changes in behavior and neurotransmission and to affect the movement, but there should be no reason for concern at the concentrations detected [38].

## 3. Materials and Methods

### 3.1. Mushroom Samples

Fruiting bodies were collected from Nur Forest Park, Mazandaran Province, Iran, N 36.579904°, E 52.045385°, in October 2021, and were deposited in the Iranian Cryptogamic Herbarium (ICH), Iranian Research Organization for Science and Technology (IROST), Tehran, Iran. The identification of the specimens was performed by MG as described earlier [15]. Fruiting bodies were lyophilized and then stored at −80 °C until further analysis.

### 3.2. Proximate Analysis

Moisture, crude protein, crude fat, fiber, ash, and carbohydrate content were determined according to the Association of Official Agricultural Chemists (1995) method [39]. The moisture content was determined by heating the fresh sample at 105 °C until a constant weight was reached. The crude protein content was estimated using the Kjeldahl method. Total fat was obtained with a Soxhlet extractor using petroleum ether as the solvent. After defatting, concentrated sulfuric acid and sodium hydroxide were used to estimate crude fiber. The samples were placed in a furnace at 600 ± 15 °C to determine the ash content. The following formulas were used to calculate total carbohydrates and energy [7]:

Total carbohydrates percentage = 100 − (moisture + protein + fat + ash) %(1)

Total energy (kcal) = 4 × (g protein + g carbohydrate) + 9 × (g fat)(2)

### 3.3. Fatty Acid Composition

Fatty acids were identified according to a modification of ISO 12966-1 at the Behesht Aein Laboratory Complex, Tehran, Iran [40]. Fatty acid methyl esters (FAMEs) (after Soxhlet extraction) were obtained through the ISO 12966-2: 2017 protocol using methanolic CH_3_NaO and H_2_SO_4_ (0.2 and 1 mol/L, respectively) [41]. The fatty acid profile was analyzed on an YL6500 GC instrument (YOUNGIN Chromass, Anyang-si, Republic of Korea) with a flame ionization detector (FID) and TR-CN100 Teknokroma column. The oven temperature program was as follows: temperature change from 120 °C to 240 °C with a speed of 4 °C per minute, then held at 240 °C for seven minutes. The carrier gas (hydrogen) flow rate was one mL/min with column head pressure of 220 kPa and a split ratio of 1:100. Then, 1 µL of the sample was injected into the GC at 250 °C (injector temperature). Identification of FAMEs was performed by comparing their retention times with those of known standards.

### 3.4. Amino Acid Composition

Amino acids were analyzed at the Behesht Aein Laboratory Complex, Tehran, Iran, according to an in-house method, including three steps: (1) Acid hydrolysis of the sample to release amino acids with HCl 6N/1% phenol. (2) Amino acid derivatization with phenyl isocyanate. (3) Isolation of the derivative obtained from the preparation stage using HPLC. The YL9100 HPLC instrument (YOUNGIN Chromass, Republic of Korea) with a UV detector and software YL Clarity (version 8.1) was used. The analytical experiment was performed under the following conditions: flow rate: 1.0 mL/min, injection volume: 20 μL, absorbance at 254 nm, analytical column: C18, 5 μm, 4.6 × 250 mm, guard column: C18, 5 μm with guard column holder, column temp: 40 °C. A standard L-amino acid kit containing 22 amino acids plus glycine (Merck, Darmstadt, Germany) was used for identification. To calculate the percentage of each amino acid, the calibration curve was plotted.

### 3.5. Vitamin Content

Vitamins D2 and E (alpha-tocopherol) were determined according to national standard protocols retrieved from DIN EN 12821:2009 and DIN EN 12822: 2012 standards at the Behesht Aein Laboratory Complex, Tehran, Iran [42,43]. Methanol (50 mL), antioxidants such as ascorbic acid (0.25 g), and KOH (5 mL of 50 g/100 mL solution) were used for saponification of 5 g of mushroom powder at 80 °C for 30 min. Extraction of tocopherols and vitamin D was carried out using *n*-hexane. For identification and quantification of vitamins, the YL9100 HPLC instrument (YOUNGIN Chromass, Republic of Korea) was used with the mobile phase consisting of methanol and water (97:3), and a flow rate of 2 mL/min. Vitamin D was measured at wavelength 265 nm and vitamin E at wavelength 292 nm, with a C18 column (250 × 4 mm, 5 μm). Compounds were identified by comparing the retention times of individual peaks in the chromatograms and the standard solutions.

### 3.6. Mineral Content

The concentrations of Na, K, Fe, Ca, P, Mg, Mn, Cu, Zn, Cr, Pb, Cd, As, and Hg in the mushroom samples were analyzed using an inductively coupled plasma-mass spectrometer (Agilent Technologies Inc.-7500 ICP-MS, Santa Clara, CA, USA). For sample preparation, 5 mL of HNO_3_ was added to 0.1 g of mushroom powder. Via indirect heating, the sample was completely dissolved in acid and the filtered solution reached a volume of 15 mL with pure water. The dilution factor was included in the final result. The ICP-MS measurements were performed using the instrumental conditions as follows: 1200 W radio frequency generator power with 24 MHz resonance frequency, argon as nebulizer and auxiliary gas with 0.8 (L/min) flow rate, argon plasma flow rate of 12.2 (L/min), 260 total (S) sample uptake time, CCD solid-state detector, and a modified Lichte spray chamber cyclonic. An internal standard of IV-STOCK-8 and calibration solution of IV-STOCK-24 (Merck KGaA, Darmstadt, Germany) was used for calibration and quality control [44]. To calculate the daily intake (DI) of toxic heavy metals (Pb, Cd, As, Hg) and their health risk index (HRI) in mushroom, the following formulas were used:

DI = Mean concentration of toxic element in mushroom (mg/kg dw) × Daily mushroom consumption (g/day)/Average body weight (kg)(3)

HRI = DI/Oral reference dose (RfDₒ)(4)

In our study, a person’s average body weight was taken as 70 kg, and each person was assumed to consume on average 30 g dry of mushrooms per day. RfDₒ represents the extent of exposure to oral contaminants during the life, and for Cd, Hg, As, and Pb, they were 0.5, 0.3, 0.3, and 4 μg/kg/day, respectively [45,46,47].

### 3.7. Analysis of Volatile Compound Profile

The volatile compound profile was determined with a headspace GC-MS analyzer. A Trace 1310 gas chromatograph equipped with split/splitless (SSL) and programmable temperature vaporizer inlets and a TSQ Duo mass spectrometer (ThermoFisher Scientific, Waltham, MA, USA) was used. A Thermo Fisher TG-624SilMS (30 m × 0.25 mm × 1.4 µm) column was used for GC analyses. Incubation of the sample vial at 90 °C for 30 min was carried out by directly weighing 100 mg of partially dried and ground material into the headspace vial. Through a heated syringe, 1000 µL of headspace were injected into the GC-MS. According to the GC program, 35 °C was held for 1 min, followed by 5 °C/min to 120 °C, and 30 °C/min to 300 °C and 1 min. The MS parameters were as follows: 43–300 m/z scan with MS Source at 280 °C. MS spectra were matched with spectra from the Adams and NIST databases.

### 3.8. Statistical Analysis

The experiments were performed in triplicate and the obtained results were expressed as mean ± standard deviation (SD). Data visualization was carried out using the OriginPro program, version 2023b.

## 4. Conclusions

This study revealed the nutritional composition and odor-contributing volatile compound profiles of *C. alborufescens*. The findings showed the crude protein content of this mushroom is more than 25% (% dw). MGS-like amino acids form the highest amount of its amino acid content, which plays an important role in the pleasant taste of this mushroom as a non-volatile compound. GC/MS analysis showed various volatile organic compounds (VOCs), predominantly aldehydes and ketones as odor-contributing substances. In compliance with the literature on other chanterelles, our study showed that *C. alborufescens* has a high ash content, related to the high presence of macro- and microelements such as potassium, calcium, and iron. All minerals were at safe levels except mercury and arsenic, which may pose a risk for humans (exceeding the recommended values).

Notably, despite the relatively low fat content of this mushroom, it has a significant amount of fat-soluble vitamins E and D and an adequate amount of linoleic acid. Overall, this study suggests that *C. alborufescens* provides an applicable amount of protein, fiber, and minerals in a low-fat form. Also, research has shown that edible mushrooms can be used as an additive to improve the nutritional profile of food due to their valuable nutritional contents [48]. Due to the high vitamin E content of this mushroom, more studies are suggested in the field, which should use *C. alborufescens* as an antioxidant additive in food.

## Figures and Tables

**Table 1:** Nutrient content of *C. alborufescens* (g/100 g dw).

	Sample Code	1F	2F	3F
Content (mean ± SD)				
Crude protein%		22.7 ± 0.0	24.9 ± 0.6	29.7 ± 0.0
Total fat%		6.08 ± 0.07	4.50 ± 0.01	6.00 ± 0.06
Total carbohydrates%		57.6 ± 0.0	58.0 ± 0.6	52.0 ± 0.0
Fiber%		11.5 ± 0.0	11.3 ± 0.0	11.2 ± 0.0
Ash%		13.5 ± 0.0	12.4 ± 0.0	12.1 ± 0.0
Total energy (kcal)		376 ± 1	372 ± 1	381 ± 1

**Table 2:** Fatty acid content of *C. alborufescens* (g/100 g fat).

Saturated Fatty Acids	Concentrations (g/100 g fat)	Unsaturated Fatty Acids	Concentrations (g/100 g fat)
C10:0	0.60 ±0.01	C14:1	0.06 ± 0.03
C12:0	0.45 ± 0.03	C16:1	0.22 ± 0.01
C14:0	0.66 ± 0.01	C18:1n9c	28.6 ± 2.0
C16:0	18.2 ± 1.2	C18:2n6c	42.0 ± 2.5
C18:0	7.70 ± 0.50	C18:3n6	0.03 ± 0.02
C20:0	0.26 ± 0.03	C18:3n3	0.38 ± 0.01
C22:0	0.24 ± 0.03	C22:2	0.54 ± 0.03
Total SFA	28.1 ± 2.0	Total UFA	91.9 ± 2.6

(C10:0) capric acid, (C12:0) lauric acid, (C14:0) myristic acid, (C16:0) palmitic acid, (C18:0) stearic acid, (C20:0) arachidic acid, (C22:0) behenic acid, (C14:1) myristoleic acid, (C16:1) palmitoleic acid, (C18:1n9c) oleic acid, (C18:2n6c) linoleic acid, (C18:3n6) γ-linolenic acid, (C18:3n3) α-linolenic acid, (C22:2) cis-13,16-docosadienoic acid, (SFA) saturated fatty acid, (UFA) unsaturated fatty acid.

**Table 3:** Amino acid content of *C. alborufescens*.

Essential Amino Acids	Percentage	Non-Essential Amino Acids	Percentage
His	0.28 ± 0.02	Glu	3.80 ± 0.10
Ile	0.51 ± 0.04	Gly	0.10 ± 0.02
Lys	0.91 ± 0.10	Arg	0.80 ± 0.02
Leu	1.20 ± 0.05	Ala	1.00 ± 0.05
Phe	0.68 ± 0.05	Tyr	0.73 ± 0.03
Met	0.21 ± 0.01	Pro	2.0 ± 0.1
Thr	0.36 ± 0.03	Asp	1.0 ± 0.2
Val	0.61 ± 0.02	Ser	2.30 ± 0.04

Ala: alanine, Arg: arginine, Asp: aspartic acid, Glu: glutamic acid, Gly: glycine, His: histidine, Ile: isoleucine, Leu: leucine, Lys: lysine, Met: methionine, Phe: phenylalanine, Ser: serine, Thr: threonine, Tyr: tyrosine, Pro: proline, Val: valine.

**Table 4:** Major and trace element concentrations of *C. alborufescens* (mg/kg dry weight).

Elements	Concentration in *C. alborufescens*	FDA DVs (mg)	*C. alborufescens* DVs (for 30 g of dw)
Na	665 ± 6	2300	19.9
K	>10,000	4700	>300
Fe	531 ± 5	18.0	15.9
Ca	4560 ± 10	1300	137
P	5860 ± 20	1250	176
Mg	1210 ± 10	420	36.2
Mn	25.3 ± 0.2	2.3	0.7
Cu	38.1 ± 0.3	0.9	1.1
Zn	82.0 ± 0.4	11	2.4
Cr	2.20 ± 0.02	0.03	0.06

FDA DVs, daily values reported by Food and Drug Administration.

**Table 5:** Heavy metal contents of *C. alborufescens* (mg/kg dw).

Elements	Concentration in *C. alborufescens*	Daily Intake (DI)	Health Risk Index (HRI)
Pb	4.09 ± 0.04	1.75	0.43
Cd	0.80 ± 0.01	0.34	0.69
As	2.40 ± 0.02	1.03	3.45
Hg	0.80 ± 0.01	0.30	1.14

**Table 6:** Composition of the volatile constituents identified via headspace GC-MS analysis and concentrations relative to the compounds.

Compound Name	t_R_^1^ [min]	ToC ^2^ [%]	WfC ^3^ [%]	RI ^4^	Prob ^5^	SI ^6^	ID ^7^
Water	1.73	48.29	-	541	86.12	818	NIST
Acetaldehyde	1.94	7.73	16.69	547	94.67	940	NIST
Propanal	3.06	0.05	0.11	577	70.32	811	NIST
Acetone	3.15	12.80	27.63	580	87.94	900	NIST
2-Methylpropanal	4.40	1.83	3.95	613	77.89	890	NIST
*n*-Butanal	5.37	2.54	5.48	639	71.17	865	NIST
2-Butanone	5.60	0.27	0.58	645	75.95	856	NIST
3-Methylbutanal	7.37	4.14	8.94	693	82.11	893	NIST
2-Methylbutanal	7.63	2.76	5.96	700	32.43	830	NIST
1-Ethenylaziridine	7.95	0.22	0.47	709	53.43	792	NIST
*n*-Pentanal	8.87	1.40	3.02	733	84.92	910	Adams
(*Z*)-3-Hepten-1-yne	9.01	0.45	0.97	737	12.41	863	NIST
2-Hexanone	10.76	0.55	1.19	784	26.04	812	NIST
3-Penten-2-one	10.86	0.07	0.15	787	45.37	777	NIST
1-Pentanol	12.01	0.78	1.68	817	67.93	914	NIST
*n*-Hexanal	12.79	5.68	12.26	837	64.00	894	NIST
Methylpyrazine	13.48	0.50	1.08	855	66.69	864	NIST
3-Methylcyclopentyl acetate	16.15	0.24	0.52	926	95.68	859	NIST
2-Heptanone	16.33	0.43	0.93	931	69.82	864	Adams
*n*-Heptanal	16.64	0.30	0.65	939	67.01	826	NIST
2,5-Dimethylpyrazine	16.71	0.24	0.52	941	37.45	819	Adams
Ethylpyrazine	16.90	0.11	0.24	946	53.37	789	NIST
2-Pentylfuran	19.04	0.99	2.14	1005	88.33	921	NIST
Benzaldehyde	19.43	0.45	0.97	1017	33.22	761	NIST
1-Octen-3-ol	19.60	0.73	1.58	1022	80.06	918	NIST
*n*-Octanal	20.29	0.14	0.30	1042	47.92	779	NIST
3-Octen-2-one	21.91	0.26	0.56	1090	42.62	860	NIST
*n*-Nonanal	23.69	0.27	0.58	1145	28.25	790	Adams
2-Butyl-2-octenal	29.72	0.17	0.37	1426	37.25	776	NIST
Isobornyl acrylate	29.81	0.22	0.47	1432	46.62	863	NIST

^1^ **t_R_**: Retention time on the TG-624SilMS GC column. ^2^ **ToC**: Concentration relative to all compounds in the headspace given in percent. ^3^ **WfC**: Concentration relative to all compounds in the water-free headspace given in percent. ^4^ **RI_exp_**: Retention index determined relative to *n*-alkanes (C10-C25). ^5^ **Prob:** Probability for the identified compound given in percent. ^6^ **SI**: Similarity index of mass spectra. ^7^ **ID**: Databases used for identification: NIST database version 2020 or Adams 4th enhanced database.

## Data Availability

Data are contained within the article.
